# Giving a health talk

**Published:** 2014

**Authors:** Detlef Prozesky

**Affiliations:** Acting Head: Department of Medical Education, Faculty of Medicine, University of Botswana, Gaborone, Botswana.

**Figure:**
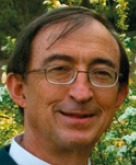
Detlef Prozesky

This is a form of health education that is used very commonly throughout the world, probably more commonly than any other method.

- Typically it takes place in a health centre or clinic, at the beginning of the day, when the patients have arrived. Health talks are also commonly given to the patients in a hospital ward.
- The subject matter is decided upon by the health care staff, often in advance – they may have a programme worked out for the month. Often the subject matter is related to the medical programme for the day – for example, if it is the day for the glaucoma clinic the talk might deal with compliance.
- The talk is usually given in the local language, which is good for communication.
- Visual aids may be used – posters, or real objects like birth control tablets.
- The popularity of health talks varies from country to country. In some countries they are not used at all, whereas in others they are used a lot. Often health centre staff members feel strongly that it is their duty to give these talks, because ‘health education’ is thought of as one of the elements of Primary Health Care. It is seen as a routine task like any other in the health centre, and is done more or less faithfully.

## The effectiveness of health talks

Health talks are a means to share information with the community, as mentioned on page 67, and this approach can support and complement more participatory approaches. There is much evidence that health talks are effective in passing on health information. Research done in South Africa in a large rural area found that the level of community knowledge about important primary health care topics was very high, and community members reported that their source of information was the local health centres.

## The advantages of health talks

- The main advantage is that you can reach a fairly large number of people at the same time; with discussions you are limited to about 10 people at a time.
- Health talks need relatively little preparation.
- Because the staff know the patients and the community, their messages are usually very relevant to the health problems and the culture of the community.

**Figure:**
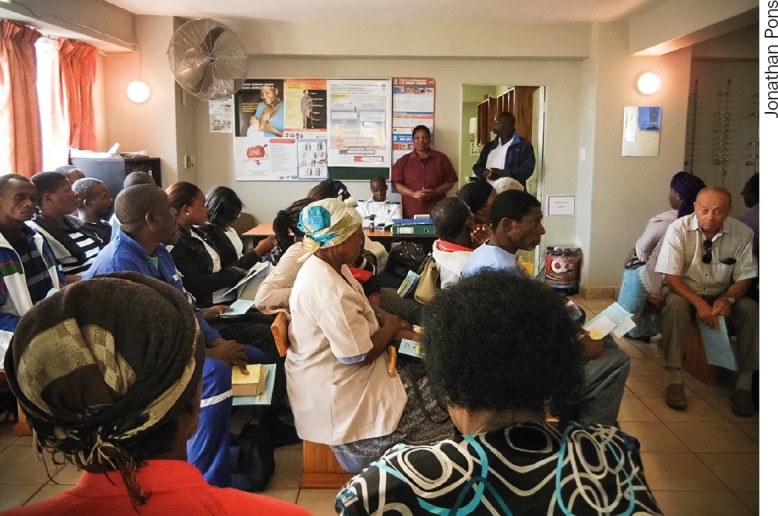
Patients listen to a health talk. SWAZILAND

## The limitations of health talks

- One of the main limitations is that you are talking to people who are already ‘converted’ to modern health care – the ones who really need the information may not come to the health centre at all.
- People may resent being kept waiting for the sake of a talk – they have their buses to catch and their lives to live.
- Only knowledge can be taught, not skills. It is also hard to empower and motivate people by just talking to them.
- Often, relatively junior staff members, with less knowledge and experience, are given the job of health education.

## Good and bad health talks

From his experience in Africa, the author has picked up the following good and bad habits that health workers have when they give health talks (see Table 1).

As in all health education, the health talk will be better if the educator addresses people's concerns and fears, deals respectfully with them, builds on useful customs that they already have, and keeps in mind their problems and limitations.

Health talks are a popular and very common method of health education. If they are done well, they can be highly effective in passing on important knowledge.

## Top tips

- Timing: Keep it to 20 minutes
- When choosing content, focus on what people must know
- Remember: your hearers are only going to remember 3–4 of the facts you tell them. Make sure these are the important ones!
- When presenting the content, avoid jargon and complicated words.

**Preparing an outline to help you**

1. A striking **introduction** that creates interest and explains what is going to happen
2. A concise **body.** There must be a logical sequence of information. Reduce information to the key points.
3. A meaningful **conclusion,** containing a summary of the key or important points.

**Table 1.** Good and bad habits when giving a health talk

Good	Bad
Two-way communication – lots of interaction with the audience Short and entertaining – one or two key messages only Practical subject matter – deals with important local health issues Visual aids used Simple, understandable language Friendly, respectful and approachable Audience is encouraged to participate and ask questions Creates a jolly atmosphere with lots of laughter and interruptions Checks that the audience has understood.	One-way lecture – only the health worker talks Long and boring- too many messages for the audience to remember Subject matter is theoretical or decided on without considering local priorities No visual aids used Using lots of technical/ English words Behaving like a schoolteacher- e.g. the audience have to stand up when they ask a question, etc. Insists on a formal atmosphere Audience silent Doesn't check for understanding.

